# Genome-wide DNA methylome analysis identifies methylation signatures associated with survival and drug resistance of ovarian cancers

**DOI:** 10.1186/s13148-021-01130-5

**Published:** 2021-07-22

**Authors:** David W. Chan, Wai-Yip Lam, Fushun Chen, Mingo M. H. Yung, Yau-Sang Chan, Wai-Sun Chan, Fangfang He, Stephanie S. Liu, Karen K. L. Chan, Benjamin Li, Hextan Y. S. Ngan

**Affiliations:** 1 Department of Obstetrics and Gynaecology, L747 Laboratory Block, LKS Faculty of Medicine, 21 Sassoon Road, Pokfulam, Hong Kong, SAR People’s Republic of China; 2 Lee’s Pharmaceutical (HK) Ltd, 1/F Building 20E, Phase 3, Hong Kong Science Park, Shatin, Hong Kong People’s Republic of China; 3 Department of Obstetrics and Gynaecology, 6/F Professorial Block, Queen Mary Hospital, Pokfulam, Hong Kong People’s Republic of China

**Keywords:** Ovarian cancer, Methylome profiling, Epigenetic, Demethylating agents, Tumor grading

## Abstract

**Background:**

In contrast to stable genetic events, epigenetic changes are highly plastic and play crucial roles in tumor evolution and development. Epithelial ovarian cancer (EOC) is a highly heterogeneous disease that is generally associated with poor prognosis and treatment failure. Profiling epigenome-wide DNA methylation status is therefore essential to better characterize the impact of epigenetic alterations on the heterogeneity of EOC.

**Methods:**

An epigenome-wide association study was conducted to evaluate global DNA methylation in a retrospective cohort of 80 mixed subtypes of primary ovarian cancers and 30 patients with high-grade serous ovarian carcinoma (HGSOC). Three demethylating agents, azacytidine, decitabine, and thioguanine, were tested their anti-cancer and anti-chemoresistant effects on HGSOC cells.

**Results:**

Global DNA hypermethylation was significantly associated with high-grade tumors, platinum resistance, and poor prognosis. We determined that 9313 differentially methylated probes (DMPs) were enriched in their relative gene regions of 4938 genes involved in small GTPases and were significantly correlated with the PI3K-AKT, MAPK, RAS, and WNT oncogenic pathways. On the other hand, global DNA hypermethylation was preferentially associated with recurrent HGSOC. A total of 2969 DMPs corresponding to 1471 genes were involved in olfactory transduction, and calcium and cAMP signaling. Co-treatment with demethylating agents showed significant growth retardation in ovarian cancer cells through differential inductions, such as cell apoptosis by azacytidine or G2/M cell cycle arrest by decitabine and thioguanine. Notably, azacytidine and decitabine, though not thioguanine, synergistically enhanced cisplatin-mediated cytotoxicity in HGSOC cells.

**Conclusions:**

This study demonstrates the significant association of global hypermethylation with poor prognosis and drug resistance in high-grade EOC and highlights the potential of demethylating agents in cancer treatment.

**Graphic abstract:**

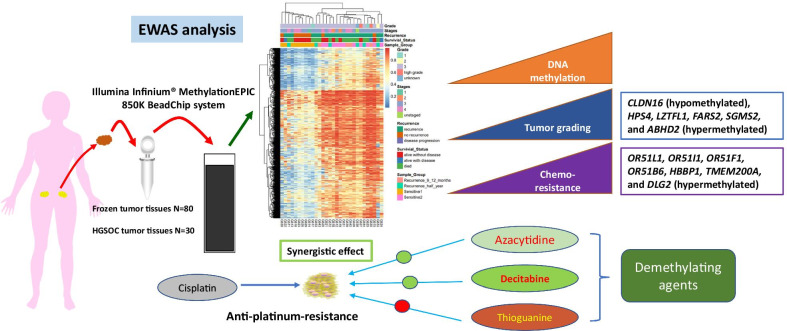

**Supplementary Information:**

The online version contains supplementary material available at 10.1186/s13148-021-01130-5.

## Background

Epithelial ovarian cancer (EOC) is one of the most lethal gynecological malignancies affecting women worldwide [1]. The high mortality rate of this disease is due to its poor prognosis and lack of reliable biomarkers, which result in the majority of EOC patients being diagnosed at an advanced stage accompanied by metastasis, chemoresistance, and high recurrence [1, 2]. EOC is a highly heterogeneous disease and consists of at least four subtypes, namely serous, clear cell, endometrioid, and mucinous, based on histopathological differences among the carcinomas [3]. Among these tumor subtypes, ~ 70% of EOC is the serous subtype followed by the endometrioid and clear cell subtypes, which account for 11–26%, and the mucinous subtype, which accounts for approximately 3% [3, 4]. Both serous and endometrioid subtypes can be classified into high-grade (HG) and low-grade (LG) EOCs [5]. Accordingly, EOC with different histological subtypes and tumor grades exhibits unique genetic mutational status, expression profiling signatures, tumorigenic properties, drug resistance, and clinical characteristics [5–7]. However, the current treatment protocols are not subtype- and/or tumor grade-specific, leading to an insufficient curative rate in this disease. Therefore, it is urgently important to better characterize the impact of genetic/epigenetic events involved in tumor heterogeneity, which may pave the way for personalized therapy.

Accumulating evidence has shown that both genetic and epigenetic alterations cooperatively drive cancer development and progression [8, 9]. However, previous studies have largely investigated genetic alterations in human cancers, while the influence of epigenetic modifications on cancer progression and development is comparatively less heavily researched [10]. Rigorous genomic evidence indicates that genetic alterations, such as somatic mutations, are stable in tumor development [8, 9]. In contrast, dynamic epigenetic changes are stimulated by endogenous or environmental factors [11, 12], indicating that the cancer epigenome is another important modulator mediating metastatic progression and the development of human cancers. DNA methylation is the most heavily studied epigenetic mechanism regulating gene expression in mammalian cells [13]. Hypermethylation of CpG islands on promoter regions leads to gene silencing, especially tumor suppressor genes (TSGs) [14]. On the other hand, global hypomethylation is also involved in oncogenesis [15]. The DNA methylation pattern is heritable but reversible and can be modulated by physiological and environmental stimuli [16, 17]. This phenomenon supports that the deregulated DNA methylation pattern is associated with cancer progression and tumor heterogeneity [18]. Hence, DNA methylation-based profiling is an additional parameter to consider for classifying tumor subtypes and achieving improved prognosis and treatment of human cancers [19, 20].

In this study, we performed an epigenome-wide association study (EWAS) of a retrospective cohort of 80 patients with mixed tumor subtypes and 30 HGSOC of primary ovarian cancers by utilizing the Illumina Infinium® MethylationEPIC BeadChip system (850 k) as the platform to interrogate over 850,000 CpG sites in the human genome. DNA methylation profiling analysis combined with clinical classifications provided a novel and in-depth understanding of the pathogenesis of EOC and the methylation signatures that may be employed in the diagnosis and treatment of this cancer.

## Materials and methods

### Study population and clinical samples

Eighty patients of epithelial ovarian cancer (EOC) with mixed tumor subtypes, stages, and grades, and 30 patients of high-grade serous subtype (HGSOC) who either survived or experienced disease recurrence after receiving first-line chemotherapy using a combination of carboplatin/paclitaxel) within 1996–2017 were randomly selected from our tissue bank in the Department of Obstetrics and Gynecology, the University of Hong Kong. All tumor tissues were collected in Queen Mary Hospital, Hong Kong, and were stored in liquid nitrogen with patient consent and prior approval of the Institutional Review Board of the University of Hong Kong/Hospital Authority Hong Kong West Cluster (HKU/HA HKW IRS) (IRS Reference Number: UW 11-298).

### Histological assessment and genomic DNA isolation

Frozen cryosections were prepared from the optimal cutting temperature (OCT) frozen blocks of the frozen tumor tissue samples. Histological assessment was subsequently performed by pathologists who conducted morphological diagnosis of tumor cell percentages on cryosections of the OCT frozen blocks that were stained with hematoxylin and eosin (H&E). Total genomic DNA was extracted from tumor tissues with > 70% tumor cell content using a QIAamp® Fast DNA Tissue Kit (cat. no. 51404) (QIAGEN) according to the manufacturer’s instructions. In general, frozen tissue samples suspended in lysis buffer containing proteinase K and RNase A were homogenized with a tissue lyser at approximately 45 Hz for 2 min. The lysates were subsequently applied to the QIAamp Mini Spin Column, and the DNA bond was washed and eluted in nuclease-free water.

### Illumina 850 K methylation assay

Whole-genome DNA methylation analysis was performed by the Centre for PanorOmic Sciences (CPOS), LKS Faculty of Medicine, the University of Hong Kong, using the Infinium MethylationEPIC microarray covering over 850,000 CpGs (Infinium MethylationEPIC BeadChip Kit, WG-317-1001). In brief, bisulfite conversion was performed on the DNA samples with a Zymo EZ DNA Methylation kit (D5001-Ver.1.2.6) according to the manufacturer’s instructions and using 500 ng DNA as input. The DNA samples were subsequently denatured, amplified, fragmented enzymatically, and hybridized to Infinium beadchips. The Infinium HD Assay Methylation Protocol Guide [15019519 v06] was followed with the following settings: the incubation time for whole genome amplification was 22 h 15 min (20–24 h in the protocol), and the hybridization time was 16 h 30 min (16–24 h in the protocol). After hybridization, the beadchips were washed to remove unhybridized and nonspecifically hybridized DNA and were subjected to allele-specific single-base extension and staining thereafter. The beadchips were imaged on Illumina iScan System. Data files were subjected to QC via GenomeStudio Version 2011.1.

### Data preprocessing and differential methylation analysis

Illumina intensity data files (.idat) from the chip were further processed by the R/Bioconductor (version 3·6·1) package ChAMP (version 2.8.6). Probes with a *P *value > 0·01 and those located on the X and Y chromosomes were filtered out. In addition, any probes with < 3 beads in at least 5% of samples per probe, non-CpG probes, SNP-related probes, and all multi-hit probes were filtered out. The beta-mixture quantile (BMIQ) method was used for type-II probe normalization. Differentially methylated positions (DMPs) between each group were detected with ChAMP. DMP function using ChAMP package. We identified differentially methylated probes (DMPs) at the significance of Benjamini–Hochberg correction with adjusted *P* < 0·05 with at least a 20% difference in the methylation level between each compared group. Additionally, differentially methylated regions (DMRs) within gene promoters or first exons were defined by ChAMP. DMR function from the ChAMP package. Benjamini–Hochberg correction with adjusted *P* < 0.05 was used to estimate significant DMRs containing at least 7 CpG probes, and two consecutive locations in the DMR are separated by less than some distance of 300 bp.

### Functional annotation and pathway enrichment analysis of genes with DMPs

The gene symbols of genes with differentially methylated probes (DMPs) were converted into the Ensembl Gene ID by org.Hs.eg.db—Bioconductor in R. Gene Ontology (GO) and KEGG enrichment analyses were performed by using the clusterProfiler package in R.

### Patient survival analysis

Kaplan–Meier survival was performed using the R package ‘survival’[21]. Significance in overall or disease-free survival was calculated using the log-rank test. Cox proportional hazards regression was performed using the function coxph () from the R package ‘survival’[22, 23].

### Cell culture

Ovarian cancer (OvCa) cells (source: female) from the ES2, PEO1, and PEO4 cell lines were purchased from American Type Culture Collection (ATCC). OVCA433 cells (source: female) were provided by Prof. G.S.W. Tsao, School of Biomedical Sciences, the University of Hong Kong. A2780s and A2780cp cells were provided by Prof. Benjamin Tsang, University of Ottawa, Canada. A2780s and A2780cp cells (source: female) were two cell lines with similar origins, while A2780cp exhibited promising cisplatin resistance. PEO1 and PEO4 were another pair, with PEO4 showing enhanced cisplatin resistance. OVCA433 and ES2 cells were cultured in Dulbecco’s modified Eagle’s medium (DMEM) with 10% (v/v) fetal bovine serum (FBS) and 1% (v/v) penicillin–streptomycin (PS). A2780s, A2780cp, PEO1, and PEO4 cells were cultured in Roswell Park Memorial Institute (RPMI) 1640 medium with 10% (v/v) FBS and 1% (v/v) PS. Cells were cultured in a humidified incubator with 5% carbon dioxide (CO_2_) at 37 °C.

### 2D cell viability assay (XTT assay)

Ovarian cancer cells were seeded onto 96-well clear flat-bottom microplates at a density of 1 × 10^4^ cells/mL in 100 µL cell culture medium. The cells were treated with cisplatin, azacytidine, decitabine, or thioguanine at different concentrations in serial dilutions for 48 h. Cell viability was determined using the XTT assay. The culture medium was removed, and XTT reagent (150 µL) was added to each well. The plate was incubated in an incubator for 4 h. The absorbance at 492 nm, which represented cell viability, was measured by a microplate reader.

### 3D cell viability assay (CellTiter-Glo® 3D cell viability assay)

Ovarian cancer cells were resuspended in serum-free DMEM/F12 (1:1) medium supplemented with 10 ng/mL human basic fibroblast growth factor (hbFGF), 20 ng/mL human epidermic growth factor (hEGF), and 5 µg/mL insulin at a cell density of 1 × 10^4^ cells/mL. The cell suspension (1000 cells in 100 µL medium) was added to ultra-low attachment Nunclon™ Sphera™ 96U microplates (Thermo) and incubated for 5 days to allow spheroid formation. The spheroids were treated with cisplatin (M2223, AbMole BioScience, Houston, TX, USA) at different concentrations in serial dilutions (0 to 80 µM) and cotreated with azacytidine (M2291, AbMole BioScience), decitabine (M2052, AbMole BioScience), or thioguanine (M3258, AbMole BioScience) at various concentrations in serial dilutions (0 to 160 µM) for 72 h. Spheroid viability after drug treatment was determined using a CellTiter-Glo® 3D cell viability assay. Equal volumes of CellTiter-Glo® 3D Reagent were added to each well. The spheroids were dissociated by being pipetted up and down multiple times. The reaction mixture (100 µL) was transferred to 96-well white flat-bottom microplates and incubated at room temperature for 30 min. The luminescence representing the viability of the spheroids was measured by a Glomax® 96 Microplate Luminometer. The synergistic or antagonistic effect of cisplatin with azacytidine, decitabine, and thioguanine was analyzed using Combenefit software.

### Annexin V-PI staining

OvCa cells were seeded into 6-well tissue culture plates at 2 × 10^5^ cells/well overnight. Next, the cells were treated with different concentrations of cisplatin, azacytidine, decitabine, and thioguanine for 48 h. Cells were trypsinized and washed with PBS three times. The cells were stained with 100 μL staining solution with Annexin V-Fluorescein and propidium iodide (PI) for 15 min in the dark. After staining, 400 μL staining buffer was added to the cells. Flow cytometry was performed using a CytoFLEX flow cytometer (Beckman-Coulter). The data were analyzed using CytExpert Software (Beckman-Coulter).

### Cell cycle analysis

Ovarian cancer (OvCa) cells were seeded on 90-mm culture dishes at 5 × 10^5^ cells/dish. The cells were starved in serum-free RPMI or DMEM overnight followed by treatment with different concentrations of cisplatin, azacytidine, decitabine, and thioguanine for 48 h. Cells were trypsinized, washed with PBS, and fixed in 70% ethanol at -20 °C for 2 h. The cells were centrifuged at 800 g for 5 min and washed with PBS three times. The cells were stained with 250 μL of 50 μg/mL PI in PBS for 15 min in the dark. After staining, 750 μL PBS was added to the cells. Flow cytometry was performed using a CytoFLEX flow cytometer. The proportion of cells in each phase of the cell cycle was determined using FlowJo software.

### Statistical analysis

Statistical analysis of global DNA methylation variation was performed using unsupervised hierarchical clustering analysis with Euclidean distances. Wilcoxon test was performed to test for significant differences in DMP methylation level between the groups. Survival curves were plotted according to the Kaplan–Meier method. The statistical significance between curves was tested using the log-rank test. On the other hand, all functional assays were performed by GraphPad Prism 6.0 (San Diego, CA, USA). All data were obtained from at least three independent experiments, each performed in triplicate. Data were analyzed by unpaired t-test, and data were expressed as the mean ± S.E.M. of at least three independent tests. *P *values < 0.05 were considered to be significant.

## Results

### Elevated epigenome-wide DNA methylation status is associated with high-grade EOC

**Fig. 1:**
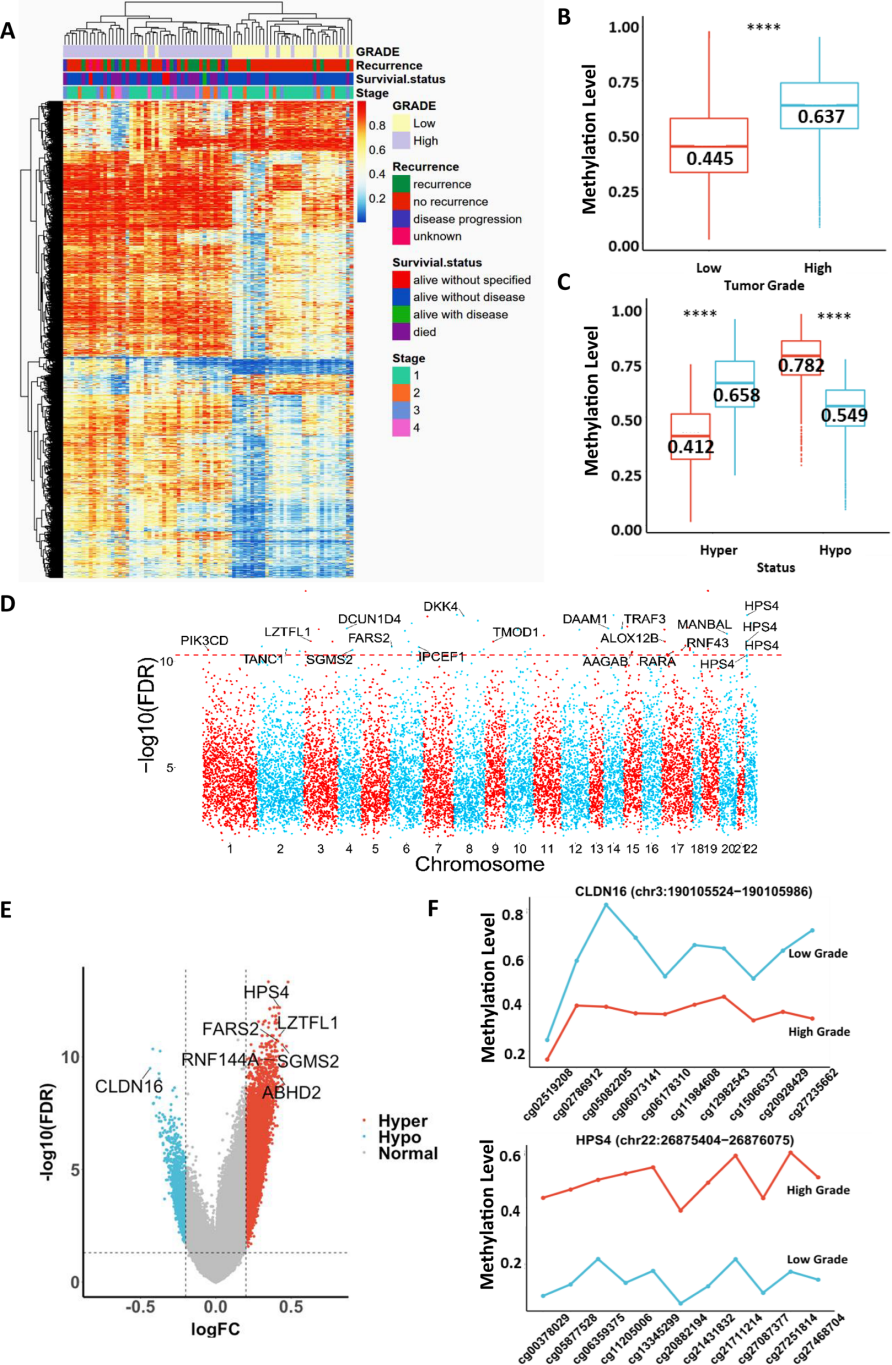
Higher global DNA methylation levels are frequently found in high-grade EOC. **a** Heatmap visualization constructed using k-means clustering of differential methylation positions (DMPs:14120) (5% false discovery rate) with at least a 20% difference in the methylation level between low- and high-grade across 80 EOC. **b** Boxplot visualization of comparison of DMP methylation levels of low- and high-grade EOC. The median DMP methylation levels were 0.445 and 0.637 in the low- and high-grade groups, respectively. *P* value (****P < 0.0001) was computed by the Wilcoxon test. **c** Boxplot visualization of comparisons of DMP methylation levels of low- and high-grade DMPs, as well as the associated hypermethylated and hypomethylated DMPs. The median value of each group is shown as a line of each box and number. *P *value (****P < 0.0001) was computed by the Wilcoxon test. **d** Manhattan plot of differentially methylated positions, where each point represents the observed − log10-adjusted *P *value at each CpG site. A total of the top 16 significant DMPs (annotated to 17 DMP-associated genes) (adjusted *P* < 5*10^−11^) found on the relevant gene promoter regions were marked. **e** Volcano plot showing the distribution of 11,986 hypermethylated DMPs (red) and 2134 hypomethylated DMPs (blue) with FDR-adjusted *P* < 0.05 and difference in *β *value (|logFC|> 0.2). Dashed lines represent cutoffs for the significant DMP-associated genes. The most differentially regulated genes of hypomethylated and hypermethylated sites are marked. (F) DMR at the promoter regions of *CLDN16* and *HPS4*. Results are plotted in comparison of DNA methylation levels of each CpG site located in the promoter regions *CLDN16* and *HPS4* in low- and high-grade EOC

This study performed EWAS using MethylationEPIC BeadChip (Illumina) to quantitatively interrogate over 850,000 CpG positions across the genome in 80 tumor tissues of mixed and pretreated EOC. The differential methylation analysis demonstrated that there was no significant correlation with tumor stages or histological subtypes of these 80 samples. Notably, there were 14,120 differentially methylated probes (DMPs) (5% false discovery rate) with at least a 20% difference in the methylation level between low-grade and high-grade EOC (|Δbeta|> 0.2) (Fig. 1a). Among 80 samples, there were three samples of grade 1, 26 samples of grade 2, 49 samples of grade 3, one sample was denoted by high grade, and one unknown tumor grading case. To improve the analysis, we omitted the unknown tumor grading case but categorized grades 1 and 2 cases as “low-grade” EOC and classified grade 3 as “high-grade” EOC. EWAS analysis demonstrated that a significantly higher content of global DNA methylation could be found in high-grade EOC (median value = 0.637) than in low-grade EOC (median value = 0.445) (Fig. 1, a, b). Among 14,120 DMPs, 11,986 (84.89%) were hypermethylated CpG sites associated with high-grade EOC (median value = 0.658), which was significantly higher than that associated with low-grade EOC (median value = 0.412) (Fig. 1c). On the other hand, 2134 out of 14,120 (15.58%) DMPs were hypomethylated CpG sites, which were strongly associated with low-grade EOC (median value = 0.782) and were significantly higher than those associated with high-grade EOC (median value = 0.549) (Fig. 1c). These findings indicate that the global DNA hypermethylation level is higher in high-grade EOC compared with low-grade EOC.

Given that global DNA hypermethylation has occurred in high-grade EOC, we were interested in analyzing the distribution of DMPs with biologically relevant on the 22 autosomal chromosomes. Our analysis indicated that the top 17 DMP-associated genes (promoter regions) were found on chromosomes 1–4, 6 8–9, 14, 15 17, 20, and 22 from 14,120 DMPs at a false discovery rate (FDR) threshold (*P* < 5*10^−11^) (Fig. 1d) (Additional file 1: Table S1). After further analysis by screening the hypermethylated or hypomethylated CpG sites within the promoter regions from the transcription start site (TSS) [24], we identified six genes, *CLDN16* (hypomethylated), *HPS4, LZTFL1, FARS2, SGMS2, ABHD2* and *RNF144A* (hypermethylated), that were potentially dysregulated and could be a novel DNA methylation signature in the classification of tumor grading (Fig. 1e). Kaplan–Meier analysis of the above-mentioned methylation signatures indicated that the hypomethylated *CLDN16* locus was positively correlated with poor overall survival (OS) in EOC (Additional file 5: Fig. S1). In contrast, the hypermethylated loci of *LZTFL1, FARS2, SGMS2, ABHD2, RNF144A* and *HPS4* were positively correlated with the poor overall survival (OS) of EOC patients (Additional file 5: Fig. S1). Indeed, the hypomethylated gene promoter regions of *CLDN16* or the hypermethylated promoter regions of *LZTFL1, FARS2, SGMS2, ABHD2, RNF144A* and *HPS4* were associated with high-grade, advanced stages (stages 3 and 4), clear cell and serous subtypes, recurrence, and high mortality rates (Additional file 6: Fig. S2). Notably, computational analysis of differentially methylated regions (DMRs) demonstrated that the levels of 10 hypomethylated CpG sites of the *CLDN16* promoter region on chr3:190105524–190105986 were significantly higher in low-grade EOC, whereas the overall levels of 11 hypermethylated CpG sites of the *HPS4* promoter region on chr22:26875404–26876075 were significantly higher in high-grade EOC (Fig. 1f). These findings indicated that the methylation status of the above-mentioned DMP-associated genes could be used as DNA methylation signatures to predict and classify aggressiveness and high- or low-grade EOC.

### Identification of signaling pathways relevant to the epigenetic signature of high-grade EOC

**Fig. 2:**
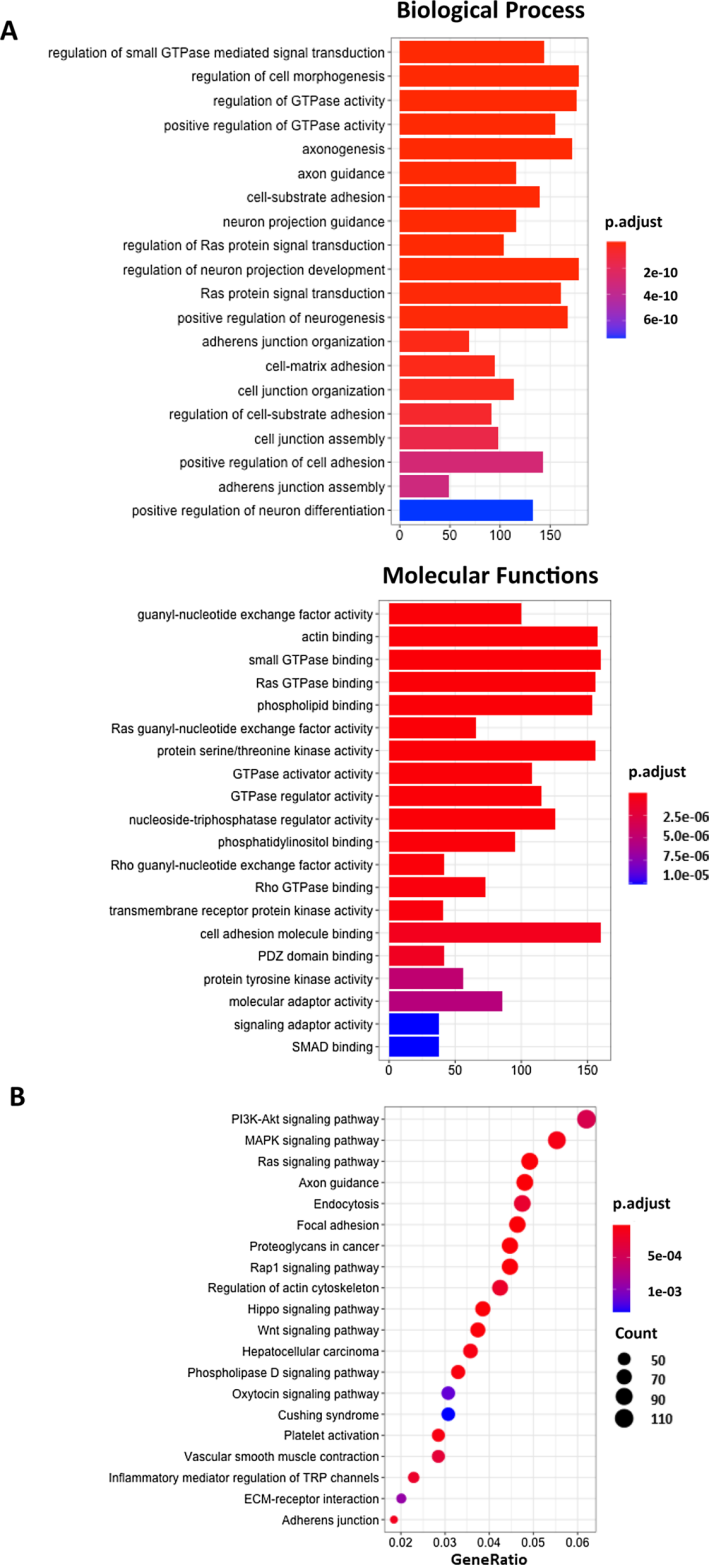
Gene ontology analyses of the differentially methylated DMPs between high- and low-grade EOC. **a** Gene ontology (GO) enrichment analysis of 4938 DMPs-associated genes enriched by 8064 hypermethylated and 1249 hypomethylated DMPs. The vertical axis represents the GO category, and the horizontal axis represents the gene count, and the color represents the p-adjusted value. **b** Kyoto Encyclopedia of Genes and Genomes (KEGG) signaling pathway enrichment of differentially methylated genes analyzed by the clusterProfiler package in R. The circle size represents gene numbers, and the color represents the *p-*adjusted value

To understand the overall functional relevance of 9313 DMPs (enriched in gene regions including promoter, gene body, 3′UTR), including 4938 genes, we performed gene ontology (GO) enrichment analysis in two annotations: biological processes and molecular functions. The results indicated that the most significantly enriched GO terms were small GTPase for signal transduction followed by RAS protein signal transduction and cell–cell signaling by WNT (Fig. 2a). In keeping with these results, among all differentially methylated genes in modulating small GTPase activities, the highest differential DMPs are enriched in their relative gene regions (promoter, gene body, 3′UTR) in relation to *TIMP2, RHOH, EphB2,* and *ARRB1,* which have been shown to be tumor suppressors and may be inhibitors of small GTPases (Additional file 2: Table S2) [25–27]. Similarly, KEGG (Kyoto Encyclopedia of Genes and Genomes) pathway analysis showed that these genes were enriched in well-known cancer pathways, such as the PI3K-AKT, MAPK, RAS, and WNT cancer signaling pathways (Fig. 2b). This enrichment was evidenced by key hypermethylated TSGs, such as *PTEN* and *PIK3R1* of the PI3K-AKT signaling pathway [28, 29], *EFNA3* and *NF1* of the MAPK signaling pathway [30, 31], *ABL1* and *PAK1* of the RAS signaling pathway [32], and *PRICKLE1* and *DAAM1* of the WNT signaling pathway [33], and these key TSGs were significantly correlated with poor OS in EOC (Additional file 7: Fig. S3, Additional file 3: Table S3). Taken together, these results indicate that hypermethylation of TSGs confers aberrant activation of oncogenic pathways involved in the aggressiveness and poor prognosis of high-grade EOC.

### Global DNA methylation increases the recurrence risk and mortality of EOC

**Fig. 3:**
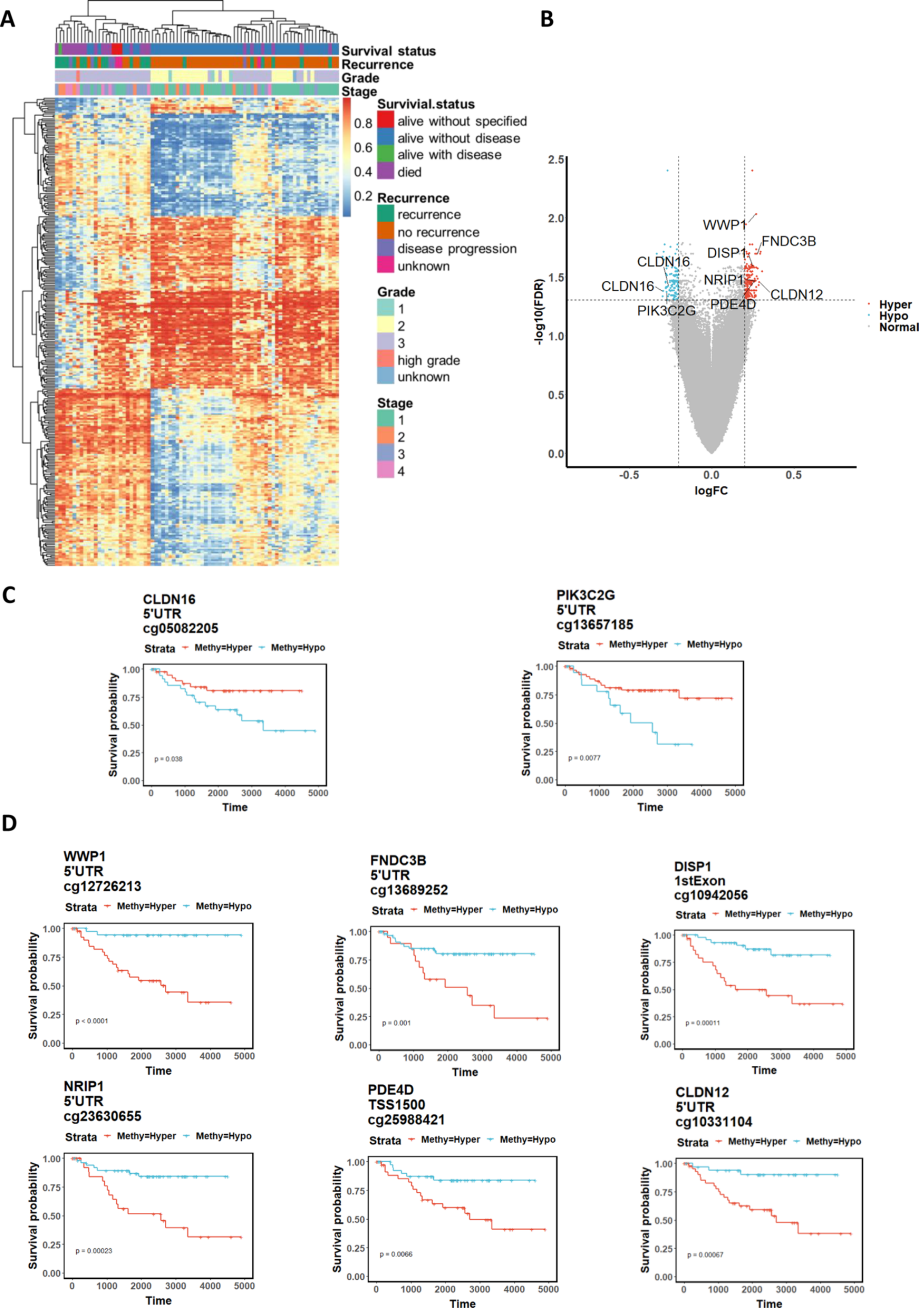
EOC with higher global DNA methylation levels has higher recurrence and poor overall survival (OS). **a** Heatmap visualization constructed using k-means clustering of differential methylation positions (DMPs:301) (5% false discovery rate) with at least a 20% difference in the methylation level between “alive without disease” and “alive with disease plus died” groups across 80 EOC cases. **b** Volcano plot showing the distribution of 180 hypermethylated DMPs (red) and 121 hypomethylated DMPs (blue) with FDR-adjusted *P* < 0.05 and difference in *β *value (|logFC|> 0.2). Dashed lines represent cutoffs for significance. The most differentially regulated genes of hypomethylated and hypermethylated sites are marked. Kaplan–Meier estimate of OS using the methylation signature of genes **c** CLDN16 and PIK3C2G) and **d** WWP1, FNDC3B, DISP1, NRIP1, PDE4D and CLDN12

High-grade EOC is usually characterized by high aggressiveness and metastatic potential [12, 34]. In this study, although our EWAS analysis could not show any significant correlation of DMPs with tumor stages, 301 DMPs are associated with a reciprocal relationship between tumor recurrence and overall survival (OS) in 80 EOC samples (Fig. 3a). Heatmap analysis of the methylation values of significant DMPs showed that EOC patients with recurrence were associated with short-term survival, and the reverse was also observed (Fig. 3a). Of 200 DMPs, we found that 177 genes had either hypomethylated or hypermethylated DMPs enriched in their relative promoter regions. The most highly differentially methylated genes enriched by DNA hypomethylated DMPs were *CLDN16* and *PIK3C2G*, while those enriched by the DNA hypermethylated DMPs were *WWP1, DISP1, FNDC3B, NRIP1, FDE4D,* and *CLDN12* (Fig. 3b). Notably, these hypomethylated and hypermethylated genes were correlated with shorter overall survival (OS) in EOC (Fig. 3c, d). These findings suggest that the DNA methylation signatures could be used to predict the disease recurrence and survival status of EOC.

### DNA methylation is associated with platinum resistance

**Fig. 4:**
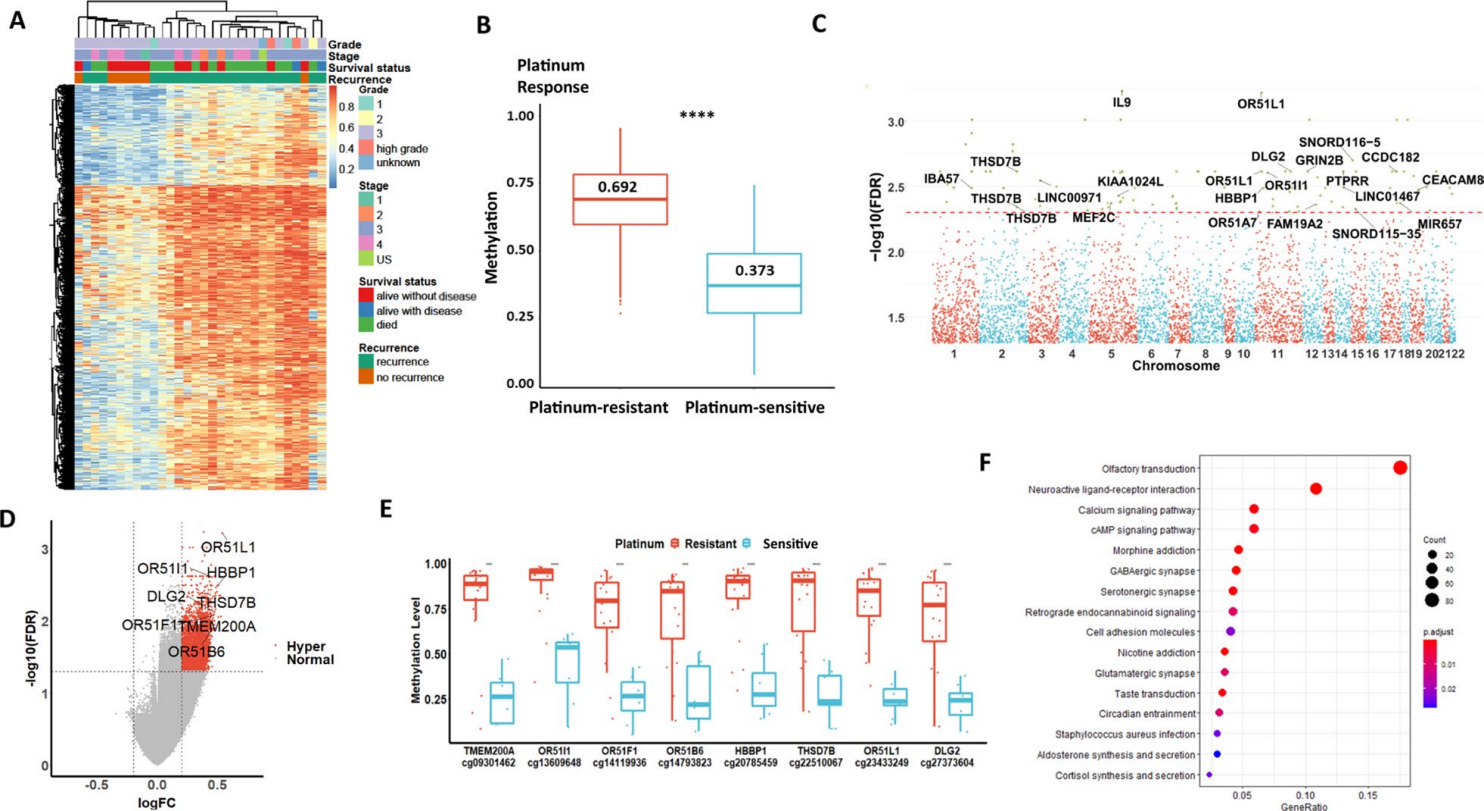
HGSOC with high DNA methylation is usually involved in high recurrence and short overall survival (OS). **a** Heatmap visualization constructed using k-means clustering of differential methylation positions (DMPs: 5844) (5% false discovery rate) with at least a 20% difference in the methylation level between the platinum- sensitive and platinum-resistant groups across the 30 HGSOC cases. **b** Boxplot visualization of comparison of the DMP methylation levels of platinum-sensitive and platinum-resistant HGSOC. The median DMP methylation levels were 0.692 and 0.373 in platinum-resistant and platinum-sensitive cells, respectively. *P *value (****P < 0.0001) was computed by the Wilcoxon test. **c** Manhattan plot of differentially methylated positions, where each point represents the observed  − log10-adjusted *P *value at a CpG site. A total of 23 significant DMPs (annotated to 20 DMP-associated genes) (adjusted *P* < 5*10^−3^) found on the relevant gene promoter regions were marked. **d** Volcano plot showing the distribution of 5844 DMP hypermethylated DMPs (red) with FDR-adjusted *P* < 0.05 and difference in *β *value (|logFC|> 0.2). Lines represent cutoff values used to identify the most hypermethylated DMPs (red). Dashed lines represent cutoffs for the significant DMP-associated genes [1471 genes]. The most differentially regulated genes of hypermethylated sites are marked. **e** Boxplot visualization of comparisons of the DNA methylation levels of platinum-resistant and platinum-sensitive groups. *P *value (****P < 0.0001) was computed by the Wilcoxon test. **f** KEGG signaling pathway enrichment of differentially methylated genes analyzed by the clusterProfiler package in R. The circle size represents gene numbers, and the color represents the *p-*adjusted value

Emerging evidence has shown that DNA methylation is involved in chemoresistance in solid cancers [35], and acquired platinum resistance is commonly observed in high-grade serous subtype ovarian cancer (HGSOC) [36]. Our EWAS indicated that 5844 DMPs could significantly show DNA methylation differences in 30 HGSOC samples (Fig. 4a). We found that 10 clinical cases belonged to the platinum-sensitive group with lower global methylation levels. Of these 10 clinical cases, 6/10 (60%) showed no recurrence within 5 years (Fig. 4a). On the other hand, the other 20 clinical cases were in the platinum-resistant group and exhibited higher global methylation levels. However, only 1 case (1/20, 5%) showed no recurrence within 5 years after receiving taxane-/platinum-based therapy as a first-line treatment (Fig. 4a). In addition, the platinum-resistant group exhibited a significantly higher content of global DNA methylation (median value = 0.692) than the platinum-sensitive group (median value = 0.373) (Fig. 4a, b). Further analysis showed that 2969 DMPs corresponding to 1471 differentially methylated genes were associated with significant DNA methylation. Among these differentially methylated genes, the top 20 DMP-associated genes (promoter regions) were found on chromosomes 1–3, 5, 11–12, 14–15, 17 and 19 at a false discovery rate (FDR) threshold (*P* < 5*10^−3^) (Fig. 4c) (Additional file 4: Table S4). Notably, the majority of DMPs were significantly associated with hypermethylated genes, including *OR51L1, OR51I1, OR51F1, OR51B6*, *HBBP1, TMEM200A,* and *DLG2* (Fig. 4d). Importantly, the DNA methylation levels of DMPs associated with these hypermethylated genes were significantly higher in the platinum-resistant group (Fig. 4e). GO enrichment analysis revealed that the most significantly differentially methylated genes were correlated with sensory perception, chemical stimulus detectors, and ion transmembrane transport regulation in biological functions, as well as ion channel activities in molecular functions (Additional file 8: Fig. S4). Additionally, KEGG enrichment analysis showed that the most enriched pathways included olfactory transduction, neuroactive ligand–receptor interaction, calcium signaling, and cAMP signaling pathways (Fig. 4f). Taken together, the results of our EWAS analysis indicated that HGSOC with higher global DNA methylation is usually involved in different levels of platinum resistance, leading to varying time intervals of recurrence after primary taxane-/platinum-based therapy.

### Demethylating agents could impair the tumor growth of ovarian cancer cells

High-grade cancers are characterized by a high potential for aggressiveness and acquired drug resistance [37]. Given that high global DNA methylation is associated with high grade and platinum resistance in EOC, the inhibition of overall DNA methylation by demethylating agents could effectively target the aggressiveness and drug resistance of EOC. To this end, we tested three commercially available demethylating reagents, azacytidine [38], decitabine [39] (suppression of DNA methyltransferase and induction of hypomethylation of DNA), and thioguanine (a purine antagonist) [40], on the viability of ovarian cancer cells. We first evaluated the IC50 values of each drug, including cisplatin, a common platinum reagent used in EOC treatment [41].

**Fig. 5:**
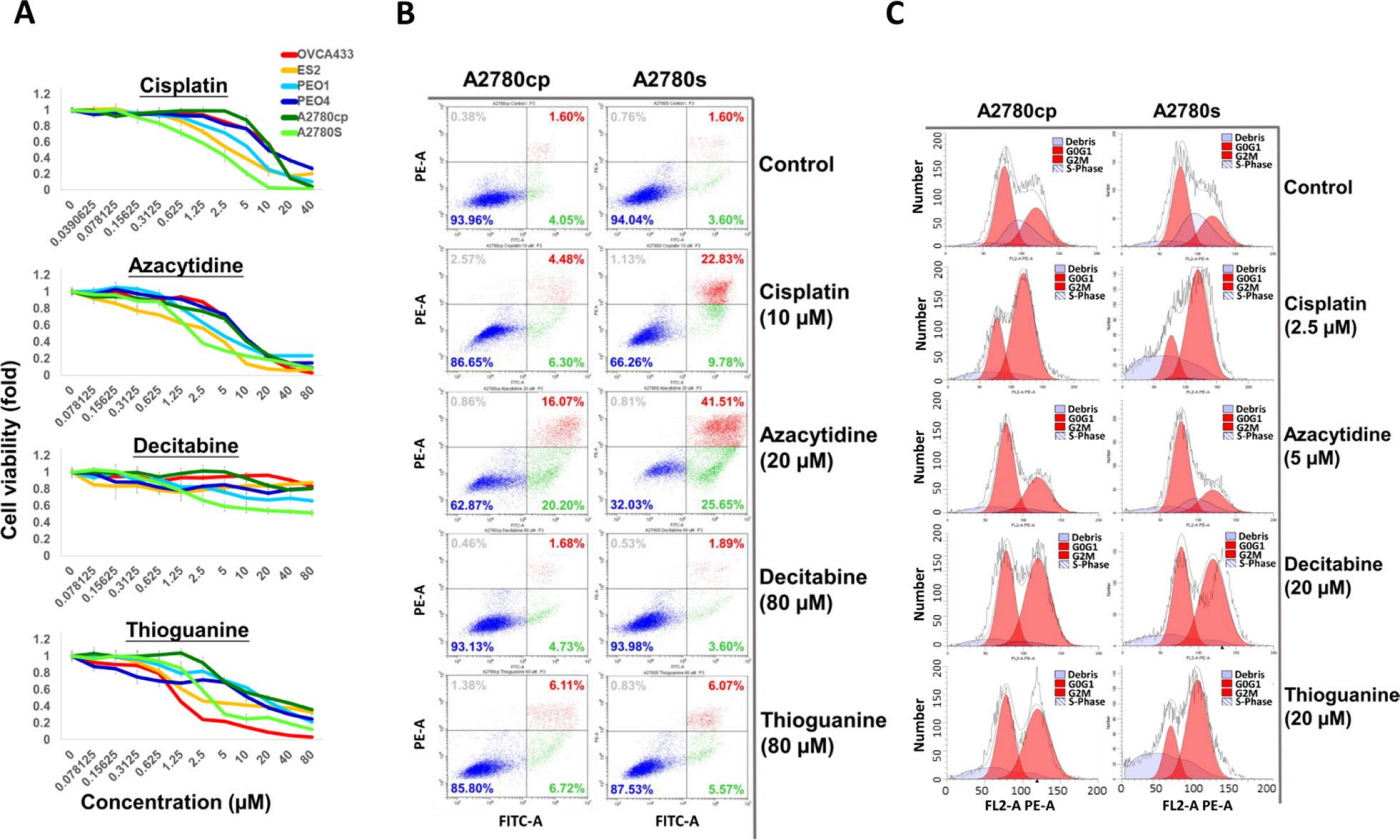
Cisplatin, azacytidine, decitabine, and thioguanine exerted inhibitory effects on ovarian cancer cell lines in vitro. **a** XTT assay demonstrated the reduction of cell viability on different ovarian cancer cell lines upon treatment with different concentrations of cisplatin, azacytidine, decitabine, and thioguanine for 48 h. **b** Flow cytometry on Annexin V-PI staining showed the effect of cisplatin (10 µM), azacytidine (20 µM), decitabine (80 µM) and thioguanine (80 µM) on the induction of apoptosis in A2780cp and A2780s cells after a 48-h treatment. Data analysis was performed with CytExpert software. Populations of cells distributed in different quadrants are presented in different colors. **c** Flow cytometry on PI staining revealed the effect of cisplatin (2.5 µM), azacytidine (5 µM), decitabine (20 µM), and thioguanine (20 µM) on interruption of cell cycle progression on A2780cp and A2780s cells after a 48-h treatment. Data analysis was performed with Modfit LT software. Populations of cells distributed in different phases are presented in different shaded peaks. For all, *n* = 3 technical replicates per sample. Data are represented as the mean ± SEM. *P < 0.05; **P < 0.01; ***P < 0.0001 (Student’s *t* test)

We tested 6 high-grade ovarian cancer cell lines, specifically OVCA33, PEO1, PEO4, ES-2, and a pair of isogenic ovarian cancer adenocarcinoma cell lines, A2780s (cisplatin-sensitive) and A2780cp (cisplatin-resistant) [42, 43]. The IC50 of cisplatin on these cell lines ranged from 1.94 µM to 11.95 µM, with A2780s cells being the most vulnerable to cisplatin cytotoxicity, while A2780cp and OVCA433 cells were considerably more resistant to cisplatin cytotoxicity (Fig. 5a, Additional file 9: Fig. S5A). The IC50 of azacytidine ranged from 1.97 µM in A2780s cells to 8.90 µM and 9.02 µM in OVCA433 and PEO4 cells, respectively (Fig. 5a) (Additional file 9: Fig. S5A). On the other hand, decitabine had mild or little inhibitory effect on all 6 cell lines, with IC50 values exceeding the maximal treatment dose at 80 µM (Fig. 5a, Additional file 9: Fig. S5A). Thioguanine performed better than decitabine and caused cytotoxicity in ovarian cancer cells; the IC50 was 1.17 µM for OVCA433 cells, which had a higher IC50 value at 20.98 µM for A2780cp cells (Fig. 5a, Additional file 9: Fig. S5A).

Next, we determined whether the cytotoxicity of cisplatin and three demethylating agents induced cell apoptosis or retarded cell division. To this end, we focused on A2780s and A2780cp because of their significant difference in drug responses to cisplatin and other demethylating reagents. By an Annexin V-PI staining assay, the flow cytometry analysis showed that the cisplatin-resistant A2780cp cells showed very little apoptosis until they were treated with 2.5 µM cisplatin, while the cisplatin-sensitive A2780s cells showed increasing apoptotic cell populations from 2.5, 10 to 40 µM (17.63%, 32.61%, and 63.41%, respectively) (Fig. 5b, Additional file 9: Fig. S5, B and C). Azacytidine also induced cell apoptosis in both cell lines, with A2780s cells being more responsive than A2780cp cells (Fig. 5b, Additional file 9: Fig. S5, B and C). Both decitabine and thioguanine had minor or no effect on apoptosis induction in A2780cp and A2780s cells (Fig. 5b, Additional file 9: Fig. S5, B and C).

Cell cycle analysis demonstrated that cisplatin could induce G2/M cell cycle arrest in both A2780cp and A2780s cells (Fig. 5c, Additional file 9: Fig. S5, D and E). The cell population of G2/M phase A2780cp cells was drastically elevated upon treatment with 0 to 2.5 and 10 µM cisplatin (32.01%, 72.72%, and 91.57%, respectively) (Fig. 5c, Additional file 9: Fig. S5, D and E). In contrast, lower doses of cisplatin from 0.625–2.5 µM were required to cause G2/M cell cycle arrest in A2780s cells by 46.76–78.12% (Fig. 5c, Additional file 9: Fig. S5, D and E). Upon azacytidine treatment, no notable cell cycle arrest could be detected in either A2780cp or A2780s cells, but a clear cytotoxic effect was observed in both cells with an increased SubG1 cell population upon higher dose treatment (Fig. 5c, Additional file 9: Fig. S5, D and E), suggesting that azacytidine could cause cell apoptosis. On the other hand, treatment of A2780cp and A2780s cells with decitabine at 0 to 20 µM led to an increase in the G2/M phase cell population from 35.03% to 56.66% and 28.38% to 57.41%, respectively, but there was no increase in the SubG1 cell population (Fig. 5c, Additional file 9: Fig. S5, D and E). Similarly, treatment with thioguanine at 0 to 20 µM induced G2/M arrest in A2780cp cells from 35.08% to 56.29%, but no change was observed in the SubG1 cell population (Fig. 5c, Additional file 9: Fig. S5, D and E), indicating that decitabine and thioguanine could cause cell cycle arrest only. Taken together, the three demethylating reagents examined in this study function differently in ovarian cancer cells. In brief, azacytidine could induce cell apoptosis without any effect on the cell cycle. In contrast, decitabine and thioguanine exhibited slight proapoptotic effects on cells, but they could impair cell division through the induction of G2/M cell cycle arrest.

### Suppression of global DNA methylation synergistically enhances platinum cytotoxicity in ovarian cancer cells

Cisplatin is widely used in cancer therapies, including the treatment of ovarian cancers [41]. However, the development of resistance toward cisplatin is a major obstacle facing the treatment of these cancers. Cotreatment of cisplatin with other drugs is an important approach to enhance the efficacy of cisplatin treatment. In this study, we performed cotreatment of cisplatin with azacytidine, decitabine, and thioguanine to evaluate the influence of the three drugs on the antitumor effect of cisplatin on HGSOC cells. Drugs were applied to 3D spheroids of ovarian cancer cells, in which the architectures and microenvironment of 3D culture exhibited higher similarity to solid tumors with layers of cells. Three HGSOC cell lines, PEO1, PEO4, and OVCA433, were chosen for testing [44, 45].

**Fig. 6:**
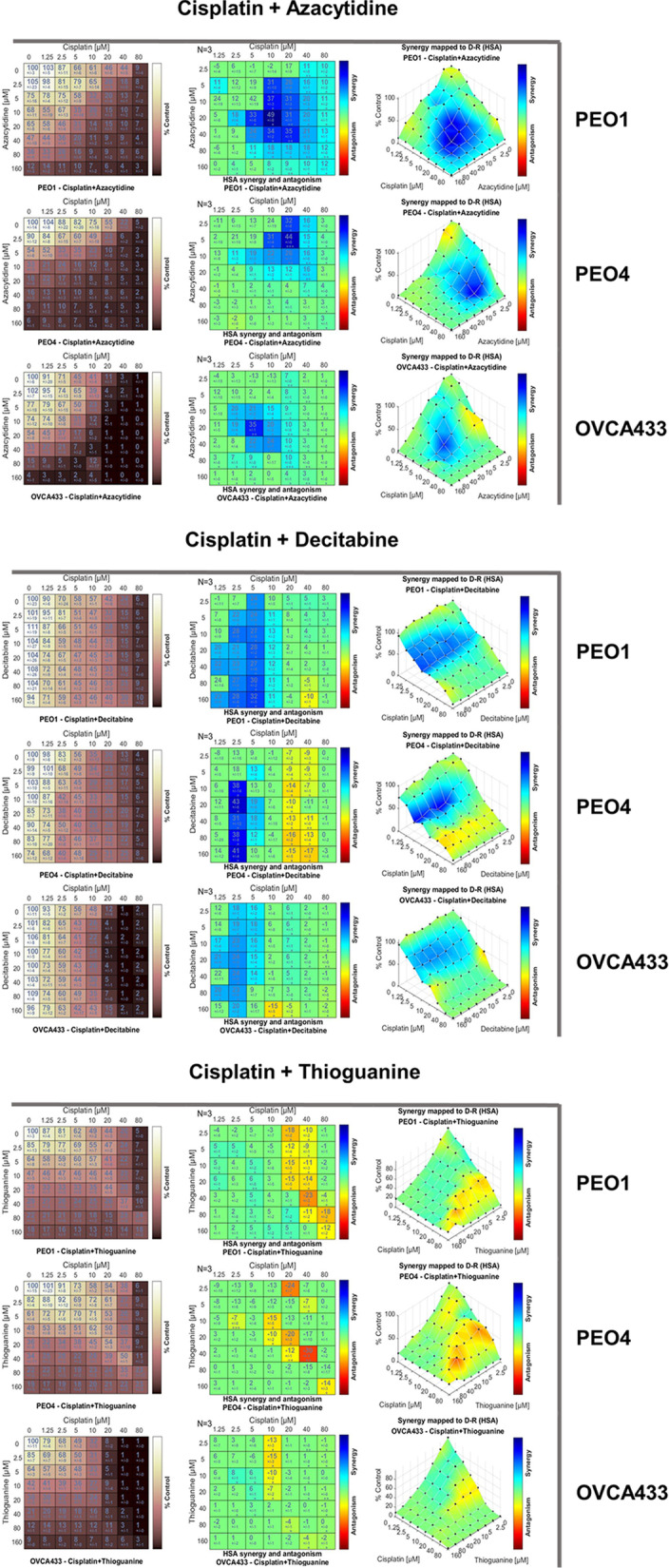
Differences in the synergistic effects of cisplatin with azacytidine, decitabine, and thioguanine were observed after cotreatment of cisplatin with the three drugs on 3D spheroids of PEO1, PEO4 and OVCA433 cells by CellTiter-Glo® 3D cell viability assay. Analysis of synergism and antagonism was performed by Combenefit analysis software using the HSA model. The viability plots, HSA synergism and antagonism plots and synergy maps are shown for each treatment on the three ovarian 3D spheroids. The regions showing increasing synergism are presented from cyan to blue, while regions showing increasing antagonism are presented from yellow to red. For all, *N* = 3 technical replicates per sample. Data are represented as the mean ± SEM. *P < 0.05; **P < 0.01; ***P < 0.0001 (Student’s *t *test)

The cell viability of PEO1, PEO4, and OVCA433 spheroids upon cotreatment of cisplatin with azacytidine, decitabine, or thioguanine was examined. Combenefit software was used to analyze synergism and antagonism with the HSA synergy score model (Fig. 6). In the HSA synergism plots of cotreatment with cisplatin and azacytidine, clear synergistic zones were available in PEO1, PEO4, and OVCA433 cells, which represented a significant synergistic effect of cisplatin and azacytidine on the suppression of ovarian cancer cell growth (Fig. 6). Under cotreatment with cisplatin and decitabine, larger synergistic zones were formed, indicating that a robust synergistic effect was achieved when using low doses of decitabine for cisplatin treatment (Fig. 6). In contrast, cotreatment with cisplatin and thioguanine led to antagonistic effects on PEO1 and PEO4, indicating that thioguanine could not cause a synergistic effect on cisplatin cytotoxicity in ovarian cancer cells (Fig. 6). These findings suggest that only azacytidine and decitabine could synergistically enhance the cytotoxic effect of cisplatin, and thioguanine does not appear to influence the antitumor effect, especially in combination with cisplatin in ovarian cancer chemotherapy.

## Discussion

The obstacles facing the clinical management of epithelial ovarian cancer (EOC) are the lack of reliable biomarkers for early diagnosis, the development of acquired drug resistance and high recurrence after treatment [1, 2]. Emerging evidence has suggested that aberrant epigenetic changes, such as DNA methylation, may represent an important mechanism governing tumor progression and drug resistance [10, 46]. Due to its dynamic nature in response to physiological and microenvironmental stimuli[47], DNA methylation has served as a potential biomarker associated with cancer diagnosis, drug resistance, and tumor progression [48, 49]. Therefore, we performed whole epigenome profiling analysis for the global methylation status of a retrospective cohort of 80 primary tumor samples of EOC. Our analysis demonstrated that global DNA hypermethylation is preferentially found in high-grade EOC, indicating that the activities of PI3K-AKT, MAPK, RAS, and WNT oncogenic pathways could be altered that may be in favor of tumor aggressiveness, shorter overall survival (OS), and a high mortality rate. In 30 HGSOC samples, a high level of global DNA methylation was observed specifically in the recurrent cases, indicating that DNA methylation is associated with acquired platinum resistance. Consistent with this result, targeting DNA methylation by using the clinically approved DNA demethylating agents such as azacytidine, decitabine, and thioguanine could notably attenuate the aggressiveness of EOC and synergistically enhance cisplatin-mediated cell cytotoxicity in HGSOC cells. Our results underscore the important role played by global DNA methylation in tumor progression and platinum resistance in EOC.

It is well known that DNA methyltransferases (DNMTs) and histone deacetylases (HDACs) are crucially involved in DNA methylation, which promotes tumor development and progression [50]. Recent evidence has shown that three DNMTs, DNMT1, DNMT3A, and DNMT3B, are frequently upregulated in aggressive EOC [51]. In keeping with these reports, our EWAS analysis demonstrated that a higher level of global DNA methylation was significantly associated with high-grade EOC, confirming that higher gene silencing events are involved in the aggressiveness of EOC. This association was supported by 9313 DMPs mapped to their relative gene regions (promoter, gene body, 3′UTR) of 4938 genes potentially silenced by DNA methylation. On the other hand, our analysis also showed hypomethylated DMPs for some genes, confirming previous reports showing that upregulated UHRF1/2 could mediate DNMT3A ubiquitination and proteasome-dependent degradation, leading to genome-wide de novo DNA methylation in cancer cells [52, 53]. However, hypermethylated DMPs were still dominant in high-grade EOC, leading to gene silencing for not only TSGs or inhibitors of small GTPases, such as *TIMP1, RHOH, EphB2,* and *ARRB1* [25–27], but also key TSGs of numerous oncogenic signaling pathways, e.g., *PTEN* and *PIK3R1* of the PI3K-AKT signaling pathway [28, 29], *EFNA3* and *NF1* of the MAPK signaling pathway [30, 31], *ABL1* and *PAK1* of the RAS signaling pathway [32], *PRICKLE1* and *DAAM1* [33]. Consistently, these aberrantly activated oncogenic pathways have been reported to promote tumor aggressiveness, drug resistance, and poor OS in high-grade EOC [54].

Our study identified 7 DMP-associated genes that could be a DNA methylation signature for determining high-grade and aggressive EOC. Of these 7 DMP-associated genes, hypomethylated *CLDN16* is a member of the claudin family modulating cell polarity and is also a biomarker of breast cancer aggressiveness [55]. Additionally, hypermethylated *LZTFL1* has been reported to be involved in maintaining cell differentiation [56], *ABHD2* was found to control anoikis resistance in HGSOC [57], *RNF144A *was shown to be a tumor suppressor in governing breast cancer growth and metastasis [58], and *HPS4* was shown to alter the functions of the small GTPases Rab32 and Rab38, which are associated with the recurrence of lung cancer [59], 60. Although another 2 genes have not been reported to have functions in cancers, these DMP-associated genes have been inferred to be putative DNA methylation signatures for predicting the high grade and aggressiveness of EOC. On the other hand, we also identified 8 DMP-associated genes that could be used to determine tumor recurrence and shorter overall survival (OS). Of these 8 DMP-associated genes, *CLDN16* and *PIK3C2G* contained DNA-hypomethylated DMPs, while *WWP1, DISP1, FNDC3B, NRIP1, FDE4D,* and *CLDN12* were enriched by DNA-hypermethylated DMPs. In the comparison of the hub gene lists, only *CLDN16* was on both lists. However, these 8 DMP-associated genes were found only from 200 DMPs encoding 177 genes, indicating that these 8 DMP-associated genes are less well-supported for use as a DNA methylation signature to predict tumor recurrence and overall survival (OS) of EOC. Further investigation using large-scale combined analyses of large-scale retrospective and prospective occurrences in EOC cohort studies is warranted.

There is an ever-growing body of evidence indicating that aberrant epigenetic alterations critically govern tumor heterogeneity, tumorigenesis, and drug resistance [61, 62]. For example, DNA methylation-mediated gene silencing of checkpoint kinase 2 (CHK2) was found to be associated with cisplatin resistance in non-small-cell lung carcinoma (NSCLC) [63]. Similarly, DNA hypermethylation of the mismatch repair gene hMSH2 was also reported in platinum-resistant EOC [64]. In fact, global DNA methylation changes leading to drug resistance have been frequently observed in various solid tumors [35]. Clinically, the responses to platinum-based chemotherapy in HGSOC can be classified into platinum-refractory, platinum-resistant and platinum-sensitive groups [65]. In this study, we excluded patients with platinum-refractory outcomes. The reasons were that this group has a complete lack of response to the first line of platinum-/taxane-based therapy, and the mechanisms for this lack of effect may include the mixed events of cancer stem cells (CSCs), genetic mutations, and epigenetic alterations [66, 67]. Indeed, this group clinically shows a significant difference from other serous subtype tumors in responding to platinum-based therapy and progression during platinum-based therapy [66]. Consistent with this finding, our EWAS found that the platinum-refractory group was different from other platinum-resistant and platinum-sensitive groups (data not shown). In contrast, our findings demonstrated that HGSOC with higher global DNA methylation usually has recurrence, implying that DNA methylation is associated with platinum resistance. In contrast to other well-known oncogenic signaling pathways in platinum resistance, such as the DNA repair pathway[68], our analysis found that the most common hypermethylation-associated genes, such as *OR51L1, OR51I1, OR51F1, OR51B6*, *HBBP1, TMEM200A, and DLG2,* are associated with olfactory transduction, neuroactive ligand–receptor interaction, calcium signaling, and cAMP signaling pathways. These pathways are very uncommon oncogenic pathways and are rarely reported in cancer drug resistance. However, emerging evidence has shown that DNA hypermethylation could affect the activities of olfactory receptor pathways regulating cancer cell proliferation and apoptosis [69], while calcium signaling and cAMP signaling pathways in association with neurotransmitters or inflammation-associated molecules could enhance ovarian cancer metastasis and drug resistance [70]. These findings suggest that further research be conducted to confirm that these DNA methylation pathways are associated with acquired drug resistance, and this study may guide new avenues of targeted therapy for recurrent HGSOC.

Targeting DNA methylation for epigenetic therapy has received increasing attention from cancer researchers [71]. Although two DNA demethylating agents, 5-azacytidine and 5-aza-2’-deoxycytidine (decitabine), have been approved for clinical use to date, hundreds of clinical trials from commercial DNA demethylating agents have examined the anticancer effect on various cancer types. However, there are accumulating examples showing variable anti-DNA methylation functions in different cancer cell types [72]. In this study, we tested three commercially available demethylating agents, azacytidine [73], decitabine [39], and thioguanine [74], on the proliferation and platinum resistance of EOC cells. As expected, these three demethylating agents could inhibit the proliferation of EOC cells by different mechanisms; azacytidine primarily enhanced cell apoptosis, while decitabine and thioguanine preferentially induced G2/M cell cycle arrest. However, only azacytidine and decitabine synergistically enhanced cisplatin-mediated cytotoxicity. This finding suggests that the selection of suitable DNA demethylating agents is very important for optimizing anticancer effects and minimizing platinum resistance.

This study is a retrospective study with a relative small sample size. The DNA methylation biomarkers identified in EWAS are still needed to have prospective validation to increase their clinical predictive value. In addition, although we have demonstrated DNA demethylating agents possess remarkable effects on anticancer and anti-drug resistance, histone modifications also closely interact with DNA demethylation in silencing gene expression in cancer progression. In future research, we should test the effect of HDAC inhibitors, such as SAHA (vorinostat) and romidepsin, on the reversibility of gene silencing and consider dual-targeted therapy.

## Conclusions

Our EWAS showed that a high level of global DNA methylation is associated with the aggressiveness of high-grade EOC and the acquired platinum resistance of HGSOC. Careful selection of demethylating agents for anti-DNA methylation is required to obtain better anticancer treatment and reduced platinum resistance in HGSOC.

## Supplementary Information

**Additional file 1:** **Table S1**. The top 17 DMP-associated genes (screened by the enrichment of DMPs on their promoter regions) on the 22 autosomal chromosomes according to the analysis of 14,120 DMPs at a false discovery rate (FDR) threshold (P < 5*10^-11^).

**Additional file 2:** **Table S2**. The list of differentially methylated genes that are involved in modulating small GTPase activities according to the 9313 DMPs in GO enrichment analysis.

**Additional file 3:** **Table S3**. According to the KEGG pathway, the list of differentially methylated genes is determined by 9313 DMPs and is involved in the PI3K-AKT, MAPK, RAS, and WNT cancer signaling pathways analysis.

**Additional file 4:** **Table S4**. The top 20 DMP-associated genes (screened by the enrichment of DMPs on their promoter regions) on the 22 autosomal chromosomes according to the analysis of 2969 DMPs at a false discovery rate (FDR) threshold (*P* < 5*10^−3^).

**Additional file 5:** **Fig. S1**. Kaplan–Meier estimate of OS using methylation signature genes (CLDN16, LZTFL1, FARS2, SGMS2, ABHD2, *RNF144A*, and HPS4).

**Additional file 6:** **Fig. S2**. Boxplot visualization of comparison of the DNA methylation level of tumor grading. EOC samples were marked by tumor stage, tumor subtype, recurrence, and survival status.

**Additional file 7:** **Fig. S3**. Heatmap visualization of DNA methylation levels in the PI3K-AKT, RAS, MAPK, and WNT signaling pathways and Kaplan–Meier estimates of OS using the methylation signature of genes.

**Additional file 8:** **Fig. S4.** Gene ontology (GO) enrichment analysis of the overall functional relevance of genes associated with DMPs using the clusterProfiler package in R.

**Additional file 9: Fig. S5.** (A) Table summarizing the IC50 values of a 48-h treatment with cisplatin, azacytidine, decitabine, and thioguanine in the six ovarian cancer cell lines using the XTT assay. IC50 values were determined at the drug concentration leading to a 50% viability reduction compared to the control. (B) Annexin V-PI staining of A2780cp and A2780s cells after a 48-h treatment with different concentrations of cisplatin, azacytidine, decitabine, and thioguanine by flow cytometry. Data analysis was performed with CytExpert software. (C) Population of apoptotic cells in each treatment condition from Annexin V-PI staining. The apoptotic cell population was represented by the cell population in the lower right quadrant (early apoptotic) and upper right quadrant (late apoptotic). (D) Cell cycle analysis of A2780cp and A2780s cells after 48 h of treatment with different concentrations of cisplatin, azacytidine, decitabine, and thioguanine by flow cytometry. Data analysis was performed with Modfit LT software. (E) Distribution of cells in different phases of the cell cycle in each treatment condition. Populations of cells distributed in G0/G1, S, G2/M and SubG1 phases are presented in different colored peaks.

## Data Availability

All methylome profiling data associated with this paper are deposited into GEO repository with the accession number GSE168930 (https://www.ncbi.nlm.nih.gov/geo/query/acc.cgi?acc=GSE168930). The other data supporting the findings of the present study are available from the corresponding author on reasonable request.

## Abbreviations

ATCC: American Type Culture Collection
CPOS: Centre for PanorOmic Sciences
DMPs: Differentially methylated probes
DMRs: Differentially methylated regions
DMEM: Dulbecco’s modified Eagle’s medium
EOC: Epithelial ovarian cancer
EWAS: Epigenome-wide association study
FBS: Fetal bovine serum
GO: Gene Ontology
hbFGF: Human basic fibroblast growth factor
hEGF: Human epidermic growth factor
HGSOC: High-grade serous ovarian carcinoma
HG: High-grade
KEGG: Kyoto Encyclopedia of Genes and Genomes
LG: Low-grade
OCT: Optimal cutting temperature
OvCa: Ovarian cancer
OS: Overall survival
PI: Propidium iodide
RPMI: Roswell Park Memorial Institute
TSGs: Tumor suppressor genes
