# Correction to: Genome-wide DNA methylome analysis identifies methylation signatures associated with survival and drug resistance of ovarian cancers

**DOI:** 10.1186/s13148-021-01141-2

**Published:** 2021-08-03

**Authors:** David W. Chan, Wai-Yip Lam, Fushun Chen, Mingo M. H. Yung, Yau-Sang Chan, Wai-Sun Chan, Fangfang He, Stephanie S. Liu, Karen K. L. Chan, Benjamin Li, Hextan Y. S. Ngan

**Affiliations:** 1 Department of Obstetrics and Gynaecology, L747 Laboratory Block, LKS Faculty of Medicine, 21 Sassoon Road, Pokfulam, Hong Kong, SAR People’s Republic of China; 2 Lee’s Pharmaceutical (HK) Ltd, 1/F Building 20E, Phase 3, Hong Kong Science Park, Shatin, Hong Kong People’s Republic of China; 3 Department of Obstetrics and Gynaecology, 6/F Professorial Block, Queen Mary Hospital, Pokfulam, Hong Kong People’s Republic of China

## Correction to: Clin Epigenet (2021) 13:142 10.1186/s13148-021-01130-5

Following publication of the original article [1], the authors identified an error in the result section.

The sentence currently read:

Indeed, the hypermethylated gene promoter regions of CLDN16 […].

The sentence should read:

Indeed, the hypomethylated gene promoter regions of CLDN16 […].

The original article [1] has been corrected.
