# Supplementary figures and images for: Genome-wide DNA methylome analysis identifies methylation signatures associated with survival and drug resistance of ovarian cancers
Source: Clin Epigenetics. 2021 Jul 22;13:142. doi: 10.1186/s13148-021-01130-5 (PMC8296615; doi:10.1186/s13148-021-01130-5)

Supplementary Figure S1

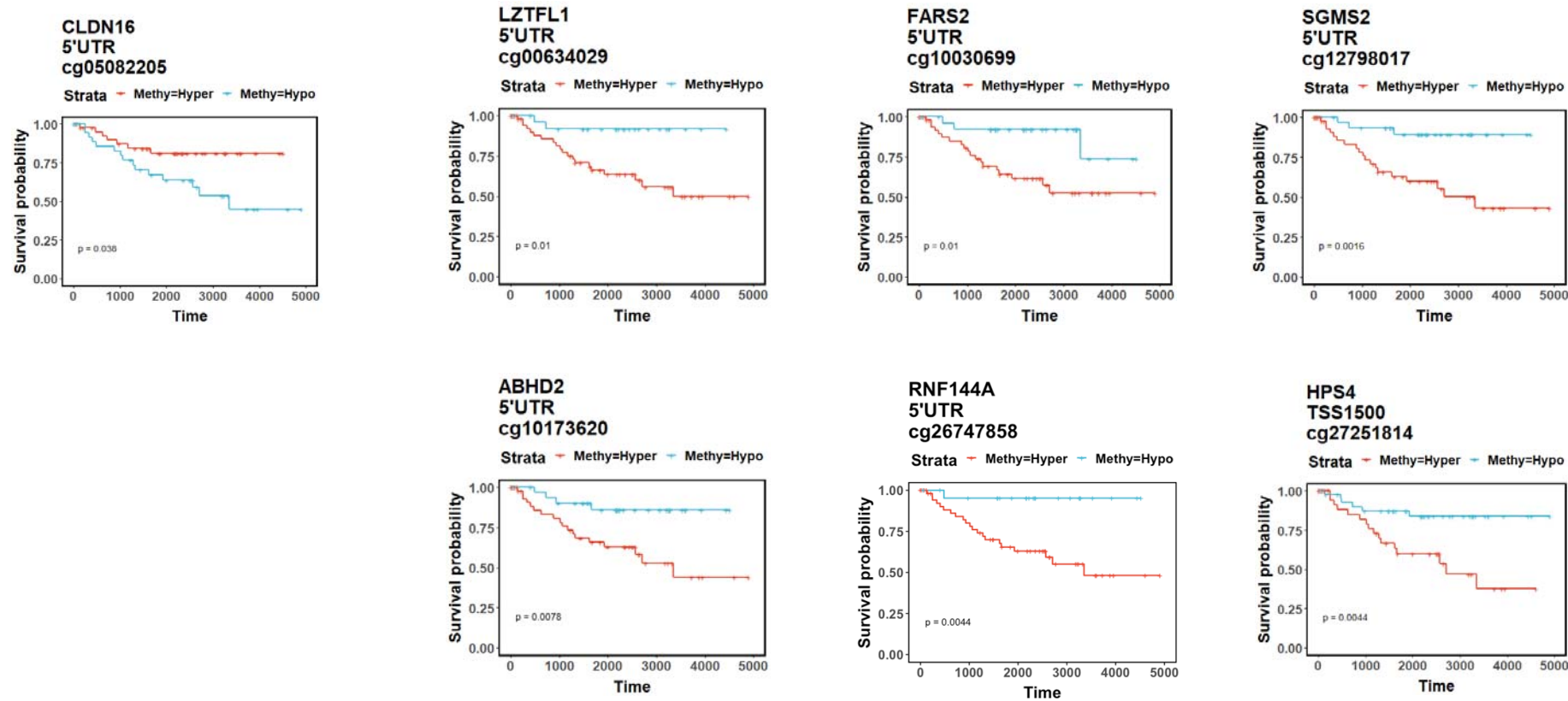

Supplement: Supplementary file 5 — Additional file 5: Fig. S1. Kaplan–Meier estimate of OS using methylation signature genes (CLDN16, LZTFL1, FARS2, SGMS2, ABHD2, RNF144A, and HPS4). [file 13148_2021_1130_MOESM5_ESM.pdf]

Supplementary Figure S2

Tumor stage

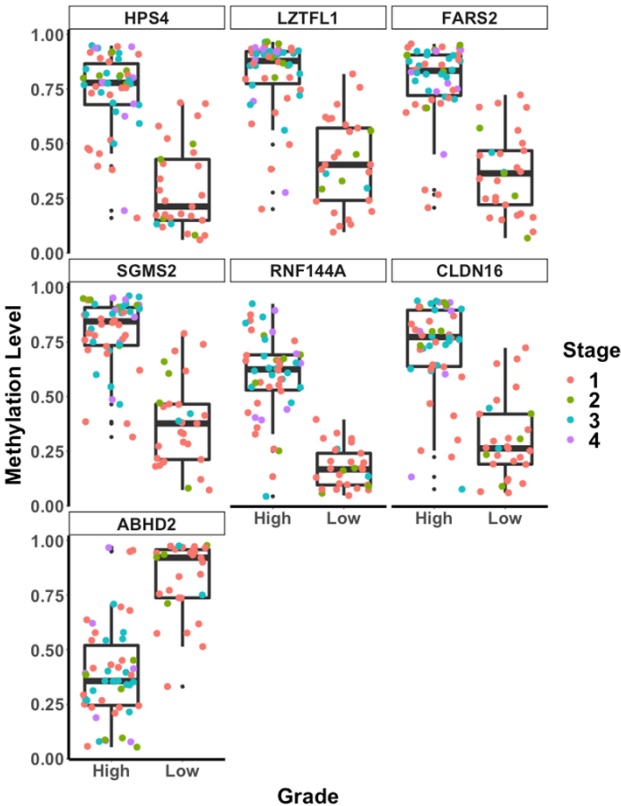

Tumor subtypes

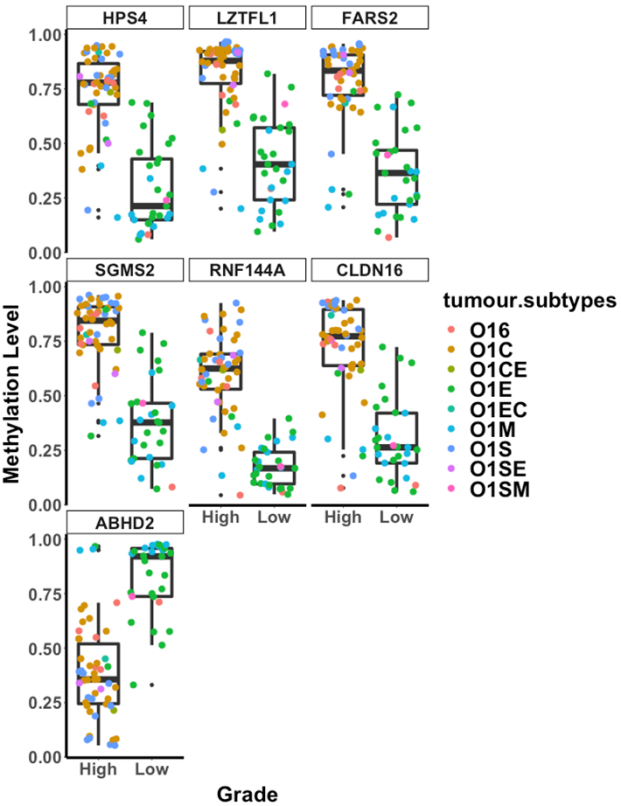

Recurrence

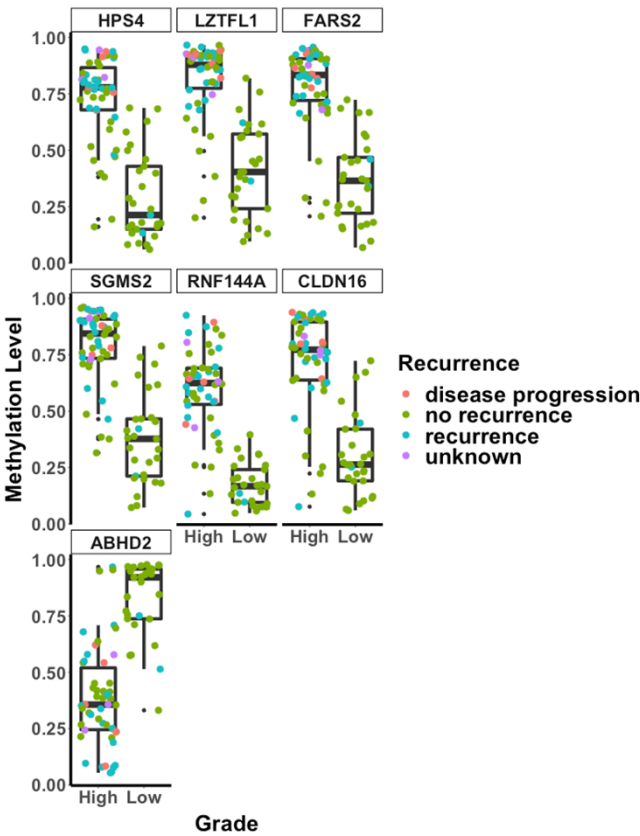

Survival status

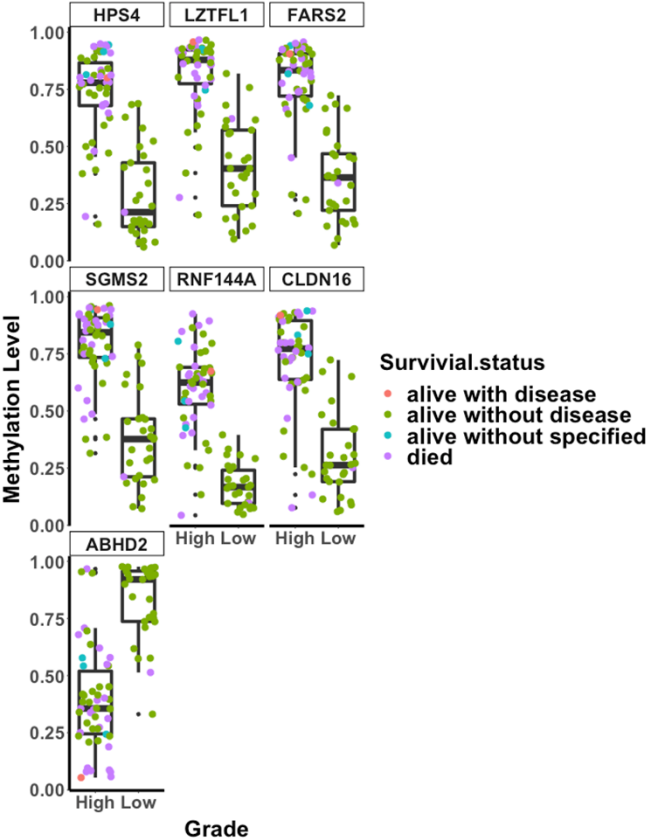

Supplement: Supplementary file 6 — Additional file 6: Fig. S2. Boxplot visualization of comparison of the DNA methylation level of tumor grading. EOC samples were marked by tumor stage, tumor subtype, recurrence, and survival status. [file 13148_2021_1130_MOESM6_ESM.pdf]

Supplementary Figure S3

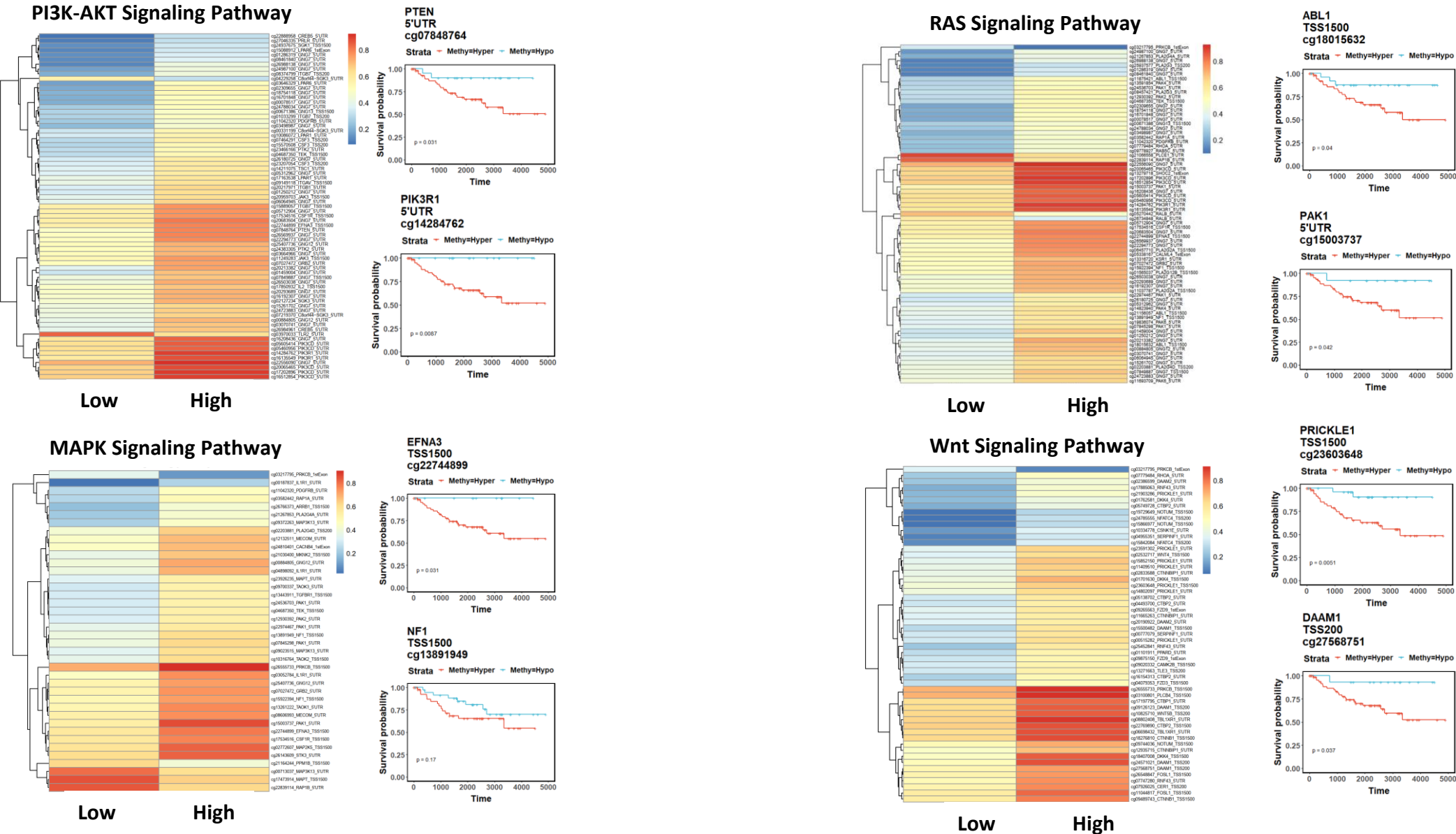

Supplement: Supplementary file 7 — Additional file 7: Fig. S3. Heatmap visualization of DNA methylation levels in the PI3K-AKT, RAS, MAPK, and WNT signaling pathways and Kaplan–Meier estimates of OS using the methylation signature of genes. [file 13148_2021_1130_MOESM7_ESM.pdf]

Supplementary Figure S4

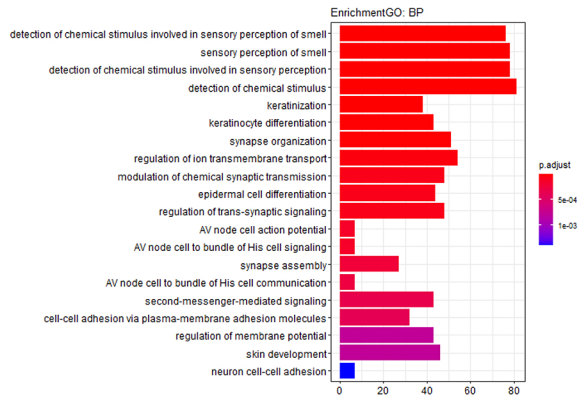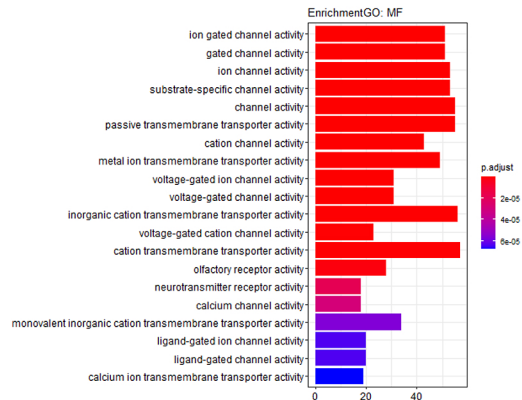

Supplement: Supplementary file 8 — Additional file 8: Fig. S4. Gene ontology (GO) enrichment analysis of the overall functional relevance of genes associated with DMPs using the clusterProfiler package in R. [file 13148_2021_1130_MOESM8_ESM.pdf]
