# Supplementary material for: Genome-wide DNA methylome analysis identifies methylation signatures associated with survival and drug resistance of ovarian cancers
Source: Clin Epigenetics. 2021 Jul 22;13:142. doi: 10.1186/s13148-021-01130-5 (PMC8296615; doi:10.1186/s13148-021-01130-5)

Supplementary Fig. S5

A

IC50 values

	Cisplatin	Azacytidine	Decitabine	Thioguanine
OVCA433	11.95 μM	8.90 μM	>80 μM	1.17 μM
ES2	3.09 μM	3.48 μM	>80 μM	2.2 μM
PEO1	5.88 μM	4.51 μM	>80 μM	17.8 μM
PEO4	9.88 μM	9.02 μM	>80 μM	10.95 μM
A2780cp	11.95 μM	8.29 μM	>80 μM	20.98 μM
A2780s	1.94 μM	1.97 μM	>80 μM	3.33 μM

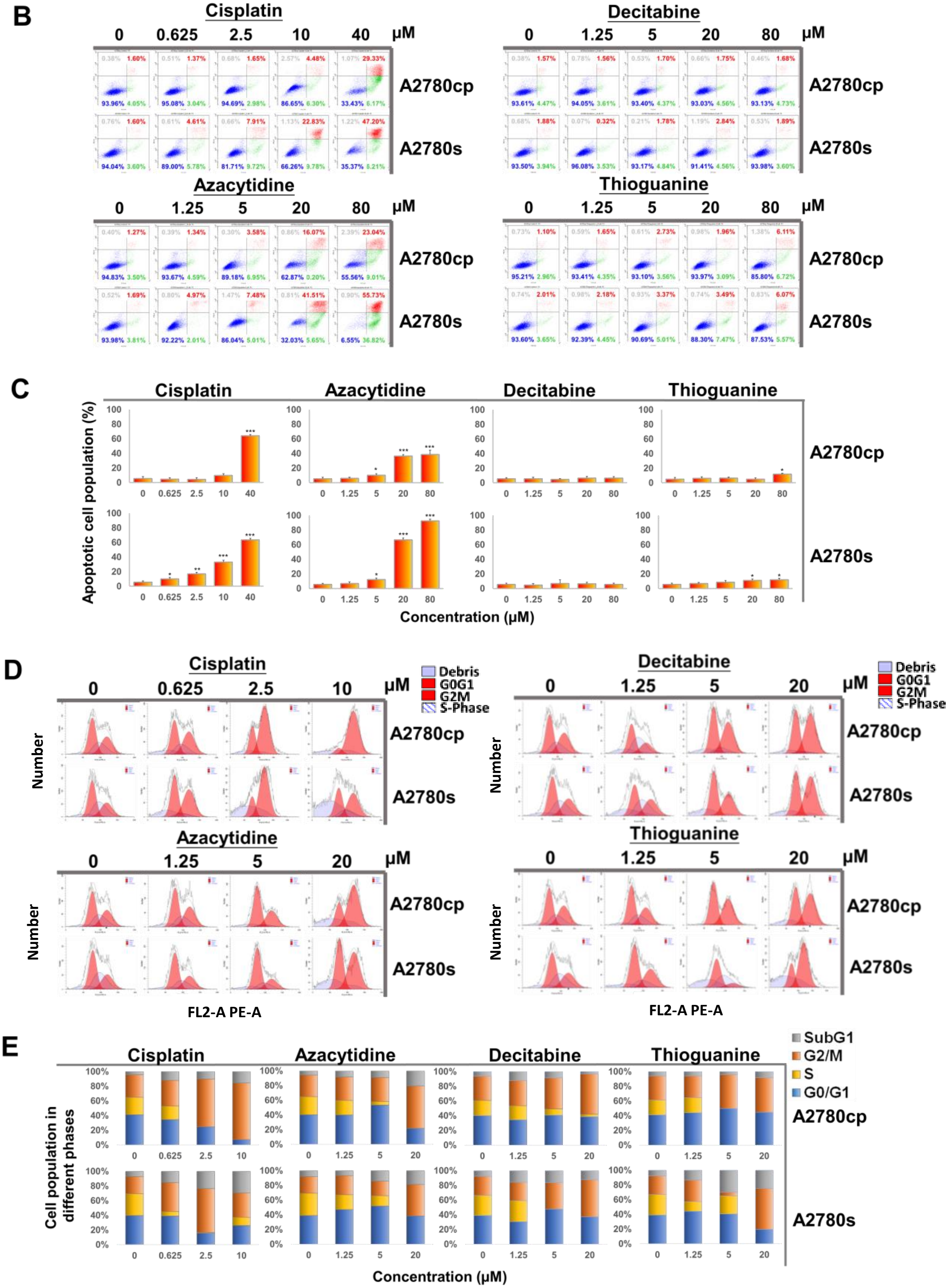

Supplement: Supplementary file 9 — Additional file 9: Fig. S5. (A) Table summarizing the IC50 values of a 48-h treatment with cisplatin, azacytidine, decitabine, and thioguanine in the six ovarian cancer cell lines using the XTT assay. IC50 values were determined at the drug concentration leading to a 50% viability reduction compared to the control. (B) Annexin V-PI staining of A2780cp and A2780s cells after a 48-h treatment with different concentrations of cisplatin, azacytidine, decitabine, and thioguanine by flow cytometry. Data analysis was performed with CytExpert software. (C) Population of apoptotic cells in each treatment condition from Annexin V-PI staining. The apoptotic cell population was represented by the cell population in the lower right quadrant (early apoptotic) and upper right quadrant (late apoptotic). (D) Cell cycle analysis of A2780cp and A2780s cells after 48 h of treatment with different concentrations of cisplatin, azacytidine, decitabine, and thioguanine by flow cytometry. Data analysis was performed with Modfit LT software. (E) Distribution of cells in different phases of the cell cycle in each treatment condition. Populations of cells distributed in G0/G1, S, G2/M and SubG1 phases are presented in different colored peaks. [file 13148_2021_1130_MOESM9_ESM.pdf]
